# Supplementary material for: Jet stream position explains regional anomalies in European beech forest productivity and tree growth
Source: Nat Commun. 2022 Apr 19;13:2015. doi: 10.1038/s41467-022-29615-8 (PMC9018849; doi:10.1038/s41467-022-29615-8)
Supplement: Supplementary file 1 — Supplementary Information [file 41467_2022_29615_MOESM1_ESM.pdf]

Supplementary Information

Jet stream position explains regional anomalies in European beech forest productivity and tree growth

Dorado Liñán *et al.*

The following Supplementary Information is available for this article:

Supplementary Tables 1 to 4

Supplementary Equation 1

Supplementary Figures 1 to 10

Supplementary Tables

Supplementary Table 1. Jet stream latitudinal position (JSL) and tree-ring width (TRW) extremes. jslPC1 and jslPC2 show the five largest positive (9th decile) and negative (1st decile) score anomalies (D90 and D10 deciles of the period 1950-2005) for the Principal Component Analysis (PCA) performed using the latitudinal position of maximum zonal-wind speed at 300 mb (JSL) for the longitudinal window 30W to 40E. trwPC1 and trwPC2 list the five largest positive and negative score anomalies (D90 and D10 deciles) for the PCA performed with 344 European beech tree-ring width chronologies for the common period 1950-2005. Bold font indicates extremes common to JSL and TRW.

**jslPC1**		**jslPC2**		**trwPC1**		**trwPC2**	
D90	D10	D90	D10	D90	D10	D90	D10
**1952**	1969	1955	1951	**1958**	1976	**1959**	**1952**
**1958**	**1982**	**1959**	**1958**	1988	**1995**	1970	1957
**1968**	**1983**	1984	1985	**1994**	1996	**1982**	**1968**
1977	1989	2002	1992	1997	**2000**	**1983**	1981
**2000**	**1995**	2005	**1994**	2001	2004	1990	1988

Supplementary Table 2. Summary of the comparison among Linear Mixed Effects Models (LMMs) to explain radial tree growth as a function of July-August JSL. Predictors include the scores of the first and second jslPCs and their one-year lagged series (Supplementary Figure 2) and geographical parameters (longitude, latitude, and elevation) of European beech forests. Standardized coefficients for the excluded predictor are derived from the full model containing all seven predictors and shown as model estimates (β) ± standard error (SE). ΔAICc indicates the relative importance of a given predictor and is calculated as the difference of AICc between the full model (i.e., including all seven predictors as fixed effects) and the model without the predictor of interest (i.e., predictor excluded). Abbreviations: scores of the leading mode of the July-August jslPCA (jslPC1); scores of the second mode of the July-August jslPCA (jslPC2); scores of the leading mode of the July-August jslPCA lagged 1 year ($jslPC1_{y-1}$); scores of the second mode of the July-August jslPCA lagged 1 year ($jslPC2_{y-1}$). Predictors displaying p-value >0.05 were excluded. Full model AICc is 54231.57.

LMM	Predictor excluded	β	SE	p-value	ΔAICc
$\beta_0 + \beta_1(jslPC2) + \beta_2(jslPC1_{y-1}) + \beta_3(jslPC2_{y-1}) + \beta_4(latitude) + \beta_5(longitude) + \beta_6(elevation)$	jslPC1	-0.085	0.007	<0.001	130.01
$\beta_0 + \beta_1(jslPC1) + \beta_2(jslPC1_{y-1}) + \beta_3(jslPC2_{y-1}) + \beta_4(latitude) + \beta_5(longitude) + \beta_6(elevation)$	jslPC2	-0.054	0.007	<0.001	53.84
$\beta_0 + \beta_1(jslPC1) + \beta_2(jslPC2) + \beta_3(jslPC2_{y-1}) + \beta_4(latitude) + \beta_5(longitude) + \beta_6(elevation)$	$jslPC1_{y-1}$	0.010	0.007	0.017	0.06
β0+ β1(jslPC1) + β2(jslPC2) + β3(jslPC1y-1) + $\beta_4(latitude) + \beta_5(longitude) + \beta_6(elevation)$	$jslPC2_{y-1}$	0.126	0.007	<0.001	295.33
$\beta_0 + \beta_1(jslPC1) + \beta_2(jslPC2) + \beta_3(jslPC1_{y-1}) + \beta_4(jslPC2_{y-1}) + \beta_5(longitude) + \beta_6(elevation)$	latitude	0.006	0.011	0.614	1.74
$\beta_0 + \beta_1(jslPC1) + \beta_2(jslPC2) + \beta_3(jslPC1_{y-1}) + \beta_4(jslPC2_{y-1}) + \beta_5(latitude) + \beta_6(elevation)$	longitude	-0.008	0.007	0.296	0.91
$\beta_0 + \beta_1(jslPC1) + \beta_2(jslPC2) + \beta_3(jslPC1_{y-1}) + \beta_4(jslPC2_{y-1}) + \beta_5(longitude) + \beta_6(latitude)$	elevation	-0.001	0.012	0.916	-1.99

Supplementary Table 3. Summary of the comparison among Linear Mixed Effects Models (LMMs) testing the exclusion of predictors as fixed effects. Standardized coefficients for the excluded predictor are derived from the full model containing all three predictors and shown as model estimates (β) ± standard error (SE). ΔAIC indicates the relative importance of a given predictor and is calculated as the difference of AICc between the full model (including the three significant predictors) and the model without the predictor of interest (i.e., predictor excluded). The predictor showing ΔAICc < 2 were excluded from the model. Abbreviations as in Supplementary Table 2. Interactions between variables have been excluded since they did not significantly decrease the AICc (i.e., interactions among jslPCs) or caused multicollinearity problems (i.e., interactions between geographical variables). Full model AICc is 54227.90.

LMM	Predictor excluded	β	SE	p-value	AICc	ΔAICc
$\beta_0 + \beta_1(jslPC2) + \beta_2(jslPC2_{y-1})$	jslPC1	-0.083	0.007	<0.001	54356.37	128.47
$\beta_0 + \beta_1(jslPC1) + \beta_2(jslPC2_{y-1})$	jslPC2	-0.053	0.007	<0.001	54281.30	53.40
$\beta_0 + \beta_1(jslPC1) + \beta_2(jslPC2)$	$jslPC2_{y-1}$	0.127	0.007	<0.001	54521.64	293.74

Supplementary Table 4. Summary of the comparison of random effects for the linear mixed effects model (LMM) including the selected fixed effects (Supplementary Table 3). ANOVA results comparing various LMMs that combine various random slopes and random intercepts. Abbreviations as in Supplementary Table 2. lat: latitude; lon: longitude; ele: elevation; df: degrees of freedom. All models include an intercept as the grouping factor. Random slopes are defined as a function of that grouping factor. Bold font indicates the selected model based on the lowest AICc and absence of significant multicollinearity (Variable Inflation Factors, VIF <2).

**Random slope ($\beta_{\chi j}$)**	**df**	**Random intercept (β_{0j})**	**AICc**
jslPC1, jslPC2, jslPC2$_{y-1}$, lat, lon, ele	33	SiteID	54230.88
jslPC1, jslPC2, jslPC2$_{y-1}$, lat, lon	26	SiteID	54216.86
jslPC1, jslPC2, jslPC2$_{y-1}$, lat, ele	26	SiteID	54220.54
jslPC1, jslPC2, jslPC2$_{y-1}$, lon, ele	26	SiteID	54219.36
jslPC1, jslPC2, lon, lat, ele	26	SiteID	54227.32
jslPC1, jslPC2$_{y-1}$, lon, lat, ele	26	SiteID	54280.05
jslPC2, jslPC2$_{y-1}$, lon, lat, ele	26	SiteID	54257.24
jslPC1, jslPC2, jslPC2$_{y-1}$, lat	20	SiteID	54205.78
jslPC1, jslPC2, jslPC2$_{y-1,}$ lon	20	SiteID	54204.84
jslPC1, jslPC2, lat, lon	20	SiteID	54217.30
jslPC1, jslPC2$_{y-1}$, lat, lon	20	SiteID	54265.96
jslPC2, jslPC2$_{y-1}$, lat, lon	20	SiteID	54245.24
jslPC1, jslPC2, jslPC2$_{y-1}$	15	SiteID	54195.79
jslPC1, jslPC2, lon	15	SiteID	54205.31
jslPC1, jslPC2$_{y-1}$, lon	15	SiteID	54255.94
jslPC2, jslPC2$_{y-1}$, lon	15	SiteID	54234.95
jslPC1, jslPC2, jslPC2$_{y-1}$	11	SiteID	54198.35
jslPC1, jslPC2, jslPC2$_{y-1}$	11	SiteID	54250.62
jslPC1, jslPC2, jslPC2$_{y-1}$	11	SiteID	54227.78
jslPC1, jslPC2, jslPC2$_{y-1}$, lat, lon, ele	33	Year	48245.70
jslPC1, jslPC2, jslPC2$_{y-1}$, lat, lon	26	Year	48998.73
jslPC1, jslPC2, jslPC2$_{y-1}$, lat, ele	26	Year	49761.41
jslPC1, jslPC2, jslPC2$_{y-1}$, lon, ele	26	Year	49386.11
jslPC1, jslPC2, lat, lon, ele	26	Year	48243.93
jslPC1, jslPC2$_{y-1}$, lat, lon, ele	26	Year	48254.92
jslPC2, jslPC2$_{y-1}$, lat, lon, ele	26	Year	48245.58
jslPC1, jslPC2, lat, lon	20	Year	48994.92
jslPC1, jslPC2, lat, ele	20	Year	49754.83
jslPC1, jslPC2, lon, ele	20	Year	49383.38
jslPC1, lat, lon, ele	20	Year	48245.94
jslPC2, lat, lon, ele	20	Year	48239.94
jslPC2, lat, lon	15	Year	48993.92
jslPC2, lat, ele	15	Year	49754.47
jslPC2, lon, ele	15	Year	49380.45
**lat, lon, ele**	**15**	**Year**	**48236.12**
lat, lon	11	Year	48990.08
lat, ele	11	Year	49750.52
lon, ele	11	Year	49379.44

Supplementary Equation 1. Final linear mixed effects model with three fixed effects

$$\gamma_{ij} = \beta_0 + \beta_1 jslPC1_{ij} + \beta_2 jslPC2_{ij} + \beta_3 jslPC2_{y\text{-}1ij} + \beta_{0j} + \beta_{1j} lat_{ij} + \beta_{2j} lon_{ij} + \beta_{3j} ele_{ij} + \varepsilon_{ij}$$

$$\beta_{kj} \sim N(0, \sigma_k^2); k = 0,1,2,3$$

$$\varepsilon_{ij} \sim N(0, \sigma^2)$$

Where γ_{ij} is observation (i.e., ring width) i at the year j; β_0 is the parameter for the intercept; $\beta_1 - \beta_3$ are the estimated sensitive JSL modes (jslPC1, jslPC2, jslPC2$_{y-1}$) for observation i at year j; β_{0j} is the random intercept for "year"; $\beta_{1j} - \beta_{3j}$ are random slopes varying across the geographical position (latitude, longitude, and elevation for observation i at the year j; ε_{ij} is the residual error term. The distribution of the random coefficients (β_{kj}) and error term (ε_{ij}) are also shown.

Supplementary Figures

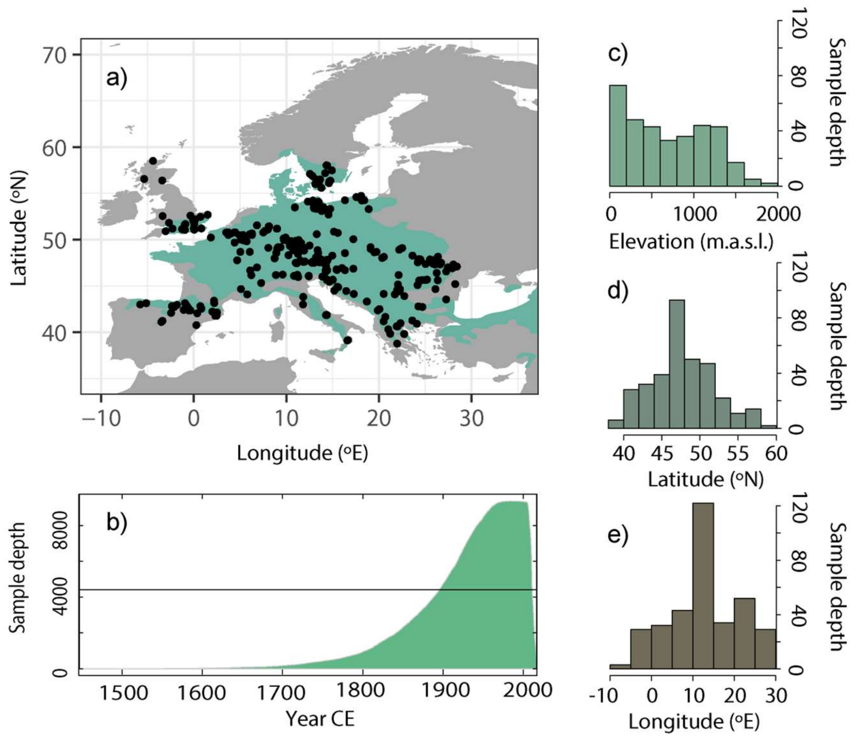

Supplementary Figure 1. Characteristics of the European Beech Tree-ring Network subset used in our study. a) Locations of the 344 study sites within the distribution range of European beech (green); b) total number of individual samples and the time-span covered; distribution of the 344 tree-ring width chronologies according to elevation (c); latitudinal position (d) and longitudinal position (e). Source of the European beech distribution data: EUFORGEN (http://www.euforgen.org)

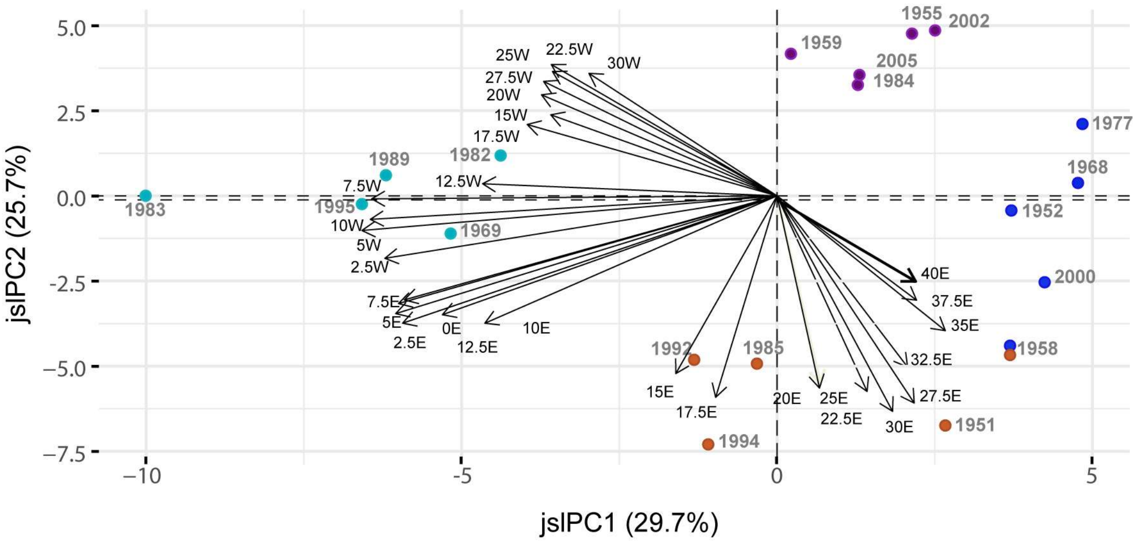

Supplementary Figure 2. Principal Component Analysis (PCA) of July-August jet stream latitude (JSL) for the common period of analyses 1950-2005. Contribution of each component, explained variance, and extreme years (i.e., D90 and D10 deciles) for the jslPC1 and jslPC2 scores are also shown: D90 of jslPC1 scores (jslPC1_D90, blue); D10 of jslPC1 scores (jslPC1_D10, cyan); D90 of jslPC2 scores (jslPC2_D90, purple); and D10 of jslPC2 scores (jslPC2_D10, orange).

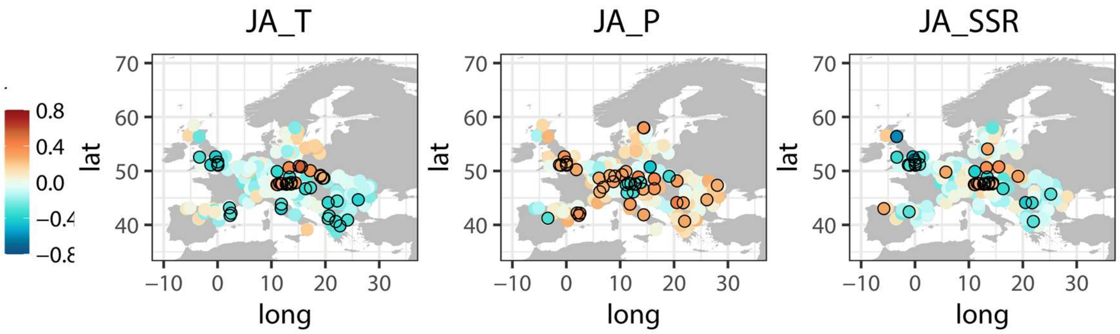

Supplementary Figure 3. Pearson's correlation coefficients between tree-ring width (TRW) chronologies and July-August air temperature (a), July-August precipitation (b), and July-August surface solar radiation (c) for the period of analysis 1950-2005. Climate data are derived from the 0.25º E-OBS gridded database[1]. Black circles indicate significant correlation between the tree-ring width series and the corresponding meteorological record ($p<0.05$).

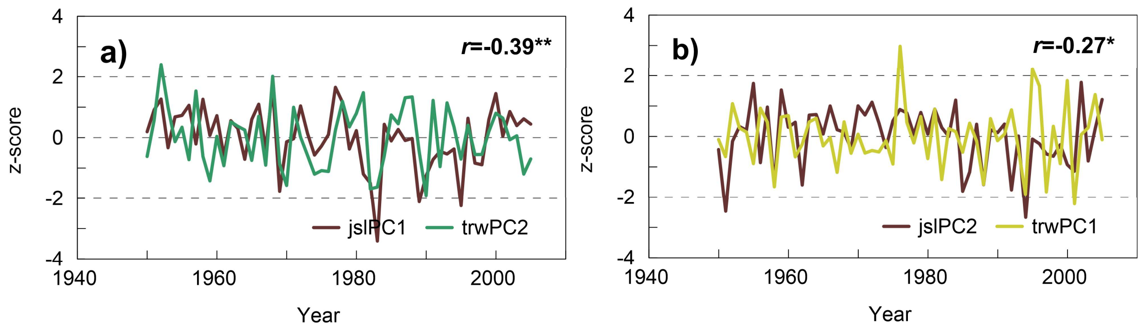

Supplementary Figure 4. Correlation between principal component (PC) scores of tree-ring width (trwPC1, trwPC2) and jsl (jslPC1 and jslPC2) for the common period of analysis 1950-2005. a) time series of jslPC1 and trwPC2 and; b) time series of jslPC2 and PC1. The Pearson's correlation coefficient between the jsl and the trw series is also shown. (*) and (**) indicate significance at $p<0.05$ and $p<0.01$. trwPC1 and trwPC2 series are reversed to improve visualization.

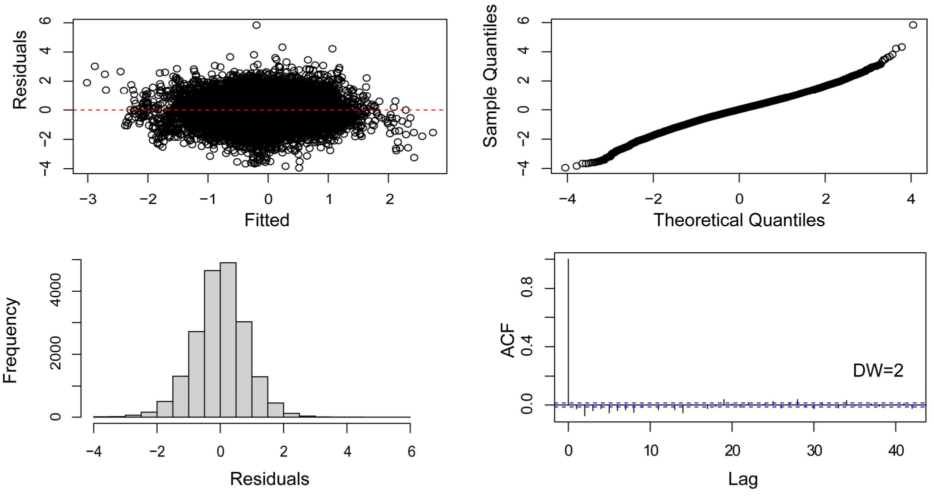

Supplementary Figure 5. Model structure and residual analysis of the linear mixed effects model (LMM) selected (Supplementary Equation 1) for explaining extreme radial tree growth anomalies as a function of jet stream latitude position (JSL). DW refers to the Durbin-Watson test.

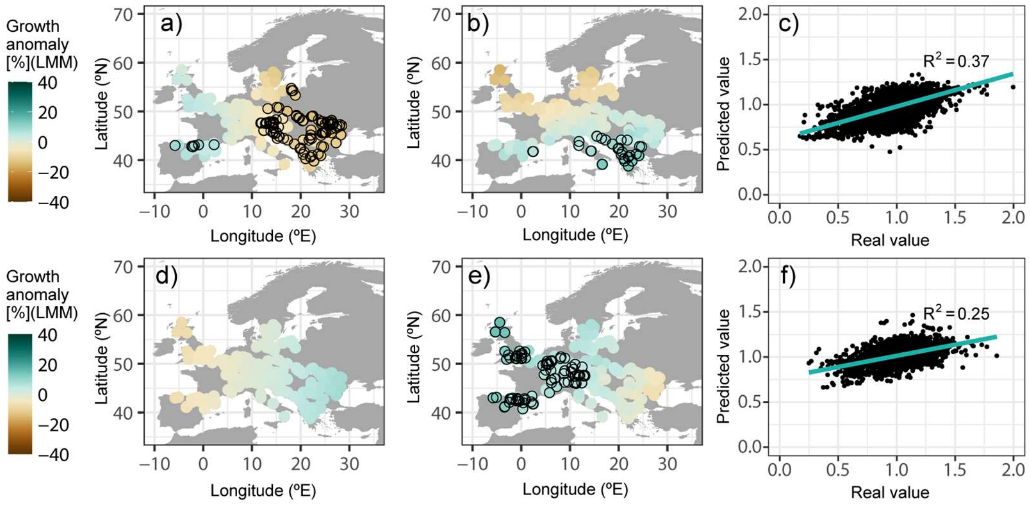

Supplementary Figure 6. Simulated European beech radial growth shown in Figure 3 using the selected LMM (Supplementary Equation 1) during summer JSL extremes. The upper panels show the simulated growth for (a) southwestern JSL displacements (jslPC1_D90) and (b) northwestern JSL displacements (jslPC1_D10), as well as the overall skills of the model (c). Lower panels show the same set of figures for southeastern and northeastern JSL displacements (i.e, jslPC2 D90 and D10, respectively). Black circles indicate significant anomalies ($p<0.05$). R^2 considering fixed and random effects (conditional R^2).

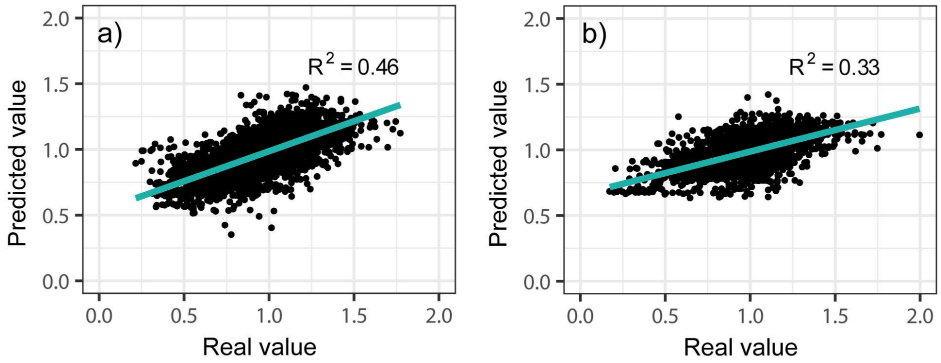

Supplementary Figure 7. Linear mixed effects model (LMM) skill for simulating the growth extremes shown in Figure 4. R^2 and overall skill of the model to simulate the extremes of the first tree-ring width mode trwPC1 (a) and the extremes of the second tree-ring width mode trwPC2 (b). R^2 considering fixed and random effects (conditional R^2).

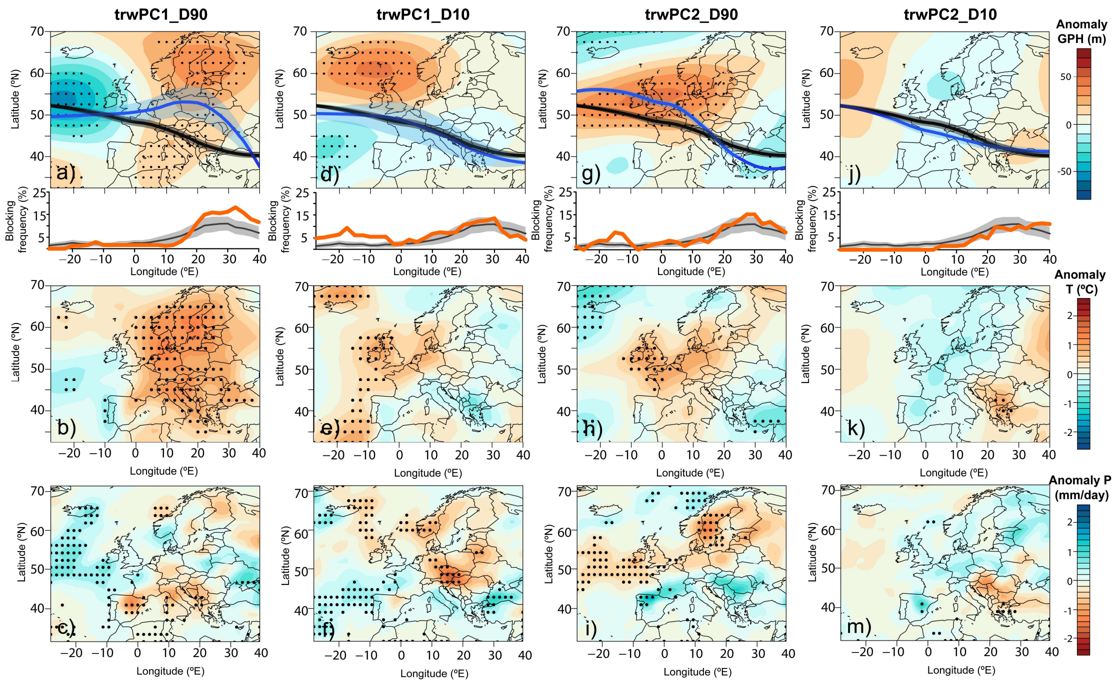

Supplementary Figure 8. July-August composite climate anomalies during tree-ring width (TRW) extreme years. Maps represent average July-August anomalies during the years of the 9th (D90) and 1st (D10) deciles of trwPC1 and trwPC2 of: 500mb geopotential height (m) (a, d, g, j), air temperature (ºC) (b, e, h, k), and precipitation (mm/day) (c, f, i, m). Black dots represent statistically significant ($p<0.05$) departures from the long-term mean climatology. Top panels also show the mean July-August JSL position between 30W-40E for the five TRW PC extreme years (blue line) and standard error (blue shading) compared to the mean for the period 1950-2005 (black lines and shading). Orange line represents mean July-August blocking frequency per longitudinal section for the five extreme years compared to the mean for the period 1950-2005 (grey line). Grey-shaded areas around the climatological mean of the blocking frequency correspond to 2 standard deviations from the mean. TrwPC1_D90 and trwPC2_D90 correspond to JSLPC2_northeastern, and JSLPC1_northwestern JSL positions, respectively (***Fig. 1j, 1d, respectively***).

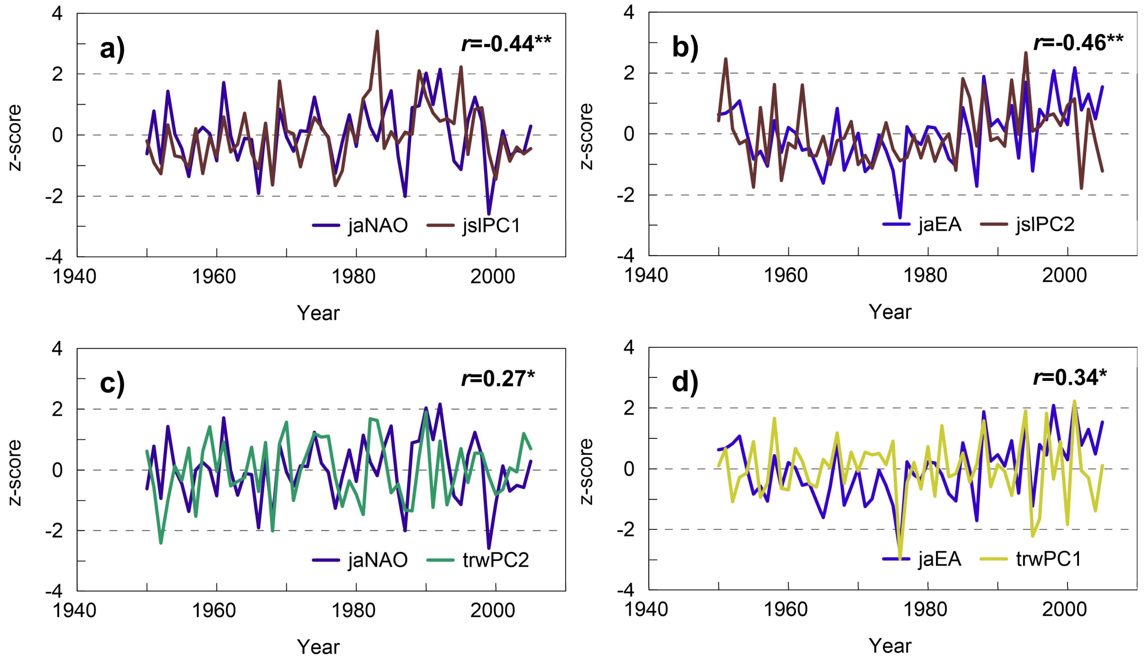

Supplementary Figure 9. Comparison between principal component scores and the main summer European atmospheric circulation patterns: the North Atlantic oscillation (NAO) and the East Atlantic pattern (EA). a) July-August jet stream latitude PC1 scores (jslPC1) and July-August NAO; b) jslPC2 and July-August EA; c) tree-ring width scores of PC1 (trwPC1) and July-August NAO; d) trwPC2 and July-August EA for the common period of analysis 1950-2005. The Pearson's correlation coefficients between the time series are also shown. (*) and (**) indicate significance at $p<0.05$ and $p<0.01$. jslPC1 and jslPC2 series reversed in upper panels to improve visualization.

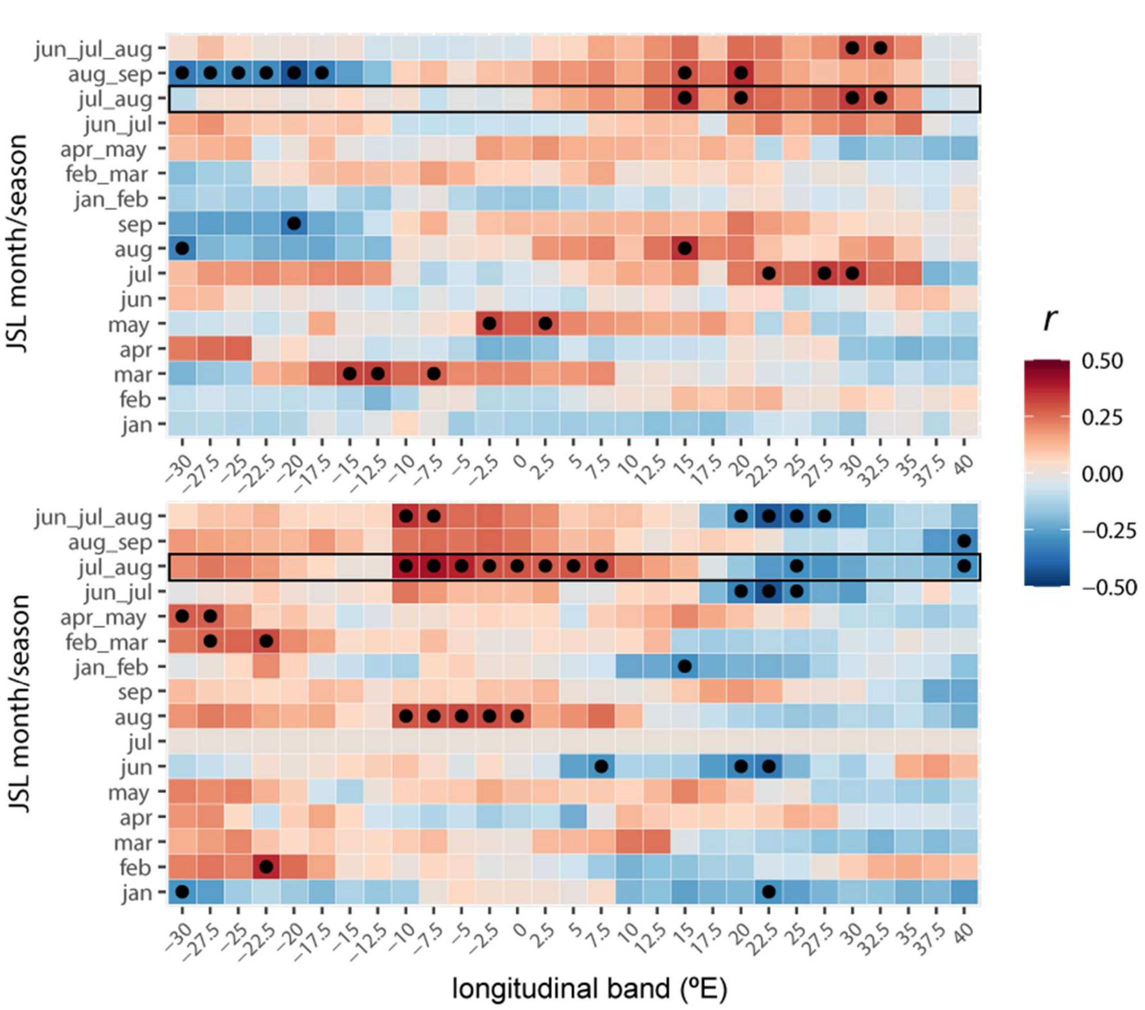

Supplementary Figure 10. Pearson's correlation coefficients between the first (trwPC1, upper panel) and second (trwPC2, lower panel) modes of radial tree growth variability and monthly and seasonal JSL per longitudinal band from 30W to 40E for the period of analysis 1950-2005. Black dots represent statistically significant correlations ($p<0.05$). The black box highlights the target JSL season July-August.

Supplementary References

1. Haylock, M. R. *et al.* A European daily high-resolution gridded data set of surface temperature and precipitation for 1950–2006. *J. Geophys. Res. Atmos.* **113**, (2008).
